# Synthesis of Silver-Coated Bioactive Nanocomposite Scaffolds Based on Grafted Beta-Glucan/Hydroxyapatite via Freeze-Drying Method: Anti-Microbial and Biocompatibility Evaluation for Bone Tissue Engineering

**DOI:** 10.3390/ma13040971

**Published:** 2020-02-21

**Authors:** Muhammad Umar Aslam Khan, Mesfer A. Al-Thebaiti, Muhammad Uzair Hashmi, Saira Aftab, Saiful Izwan Abd Razak, Shukur Abu Hassan, Mohammed Rafiq Abdul Kadir, Rashid Amin

**Affiliations:** 1 School of Biomedical Engineering, Med-X Research Institute, Shanghai Jiao Tong University (SJTU), 1954 Huashan Road, Shanghai 200030, China; umar-786@sjtu.edu.cn; 2 School of Biomedical Engineering and Health Sciences, Faculty of Engineering, Universiti Teknologi Malaysia, Skudai 81300, Malaysia; rafiq@biomedical.utm.my; 3 Department of Biology, University of Hafr Al Batin, Hafar Al-batin 39524, Saudi Arabia; malthebaiti@uhb.edu.sa; 4 Department of Industrial Biotechnology, Atta ur Rahman School of Applied Biosciences, National University of Sciences and Technology, H-12, Islamabad 44000, Pakistan; uzair.hashmi@seecs.edu.pk; 5 School of Biological Sciences, University of the Punjab, Lahore 54590, Pakistan; saira.sbs@pu.edu.pk; 6 Centre for Advanced Composite Materials, Universiti Teknologi Malaysia Skudai, Skudai 81310, Malaysia; shukur@utm.my

**Keywords:** Ag-coated scaffolds, bioactive nanocomposite, osteoblast, bone tissue engineering

## Abstract

Advancement and development in bone tissue engineering, particularly that of composite scaffolds, are of great importance for bone tissue engineering. We have synthesized polymeric matrix using biopolymer (β-glucan), acrylic acid, and nano-hydroxyapatite through free radical polymerization method. Bioactive nanocomposite scaffolds (BNSs) were fabricated using the freeze-drying method and Ag was coated by the dip-coating method. The scaffolds have been characterized by Fourier transform infrared spectroscopy (FTIR), scanning electron microscopy (SEM), and X-ray diffraction analysis (XRD) to investigate their functional groups, surface morphology, and phase analysis, respectively. The pore size and porosity of all BNS samples were found to be dependent on silver concentration. Mechanical testing of all BNS samples have substantial compressive strength in dry form that is closer to cancellous bone. The samples of BNS showed substantial antibacterial effect against DH5 alpha *E. coli*. The biological studies conducted using the MC3T3-E1 cell line via neutral red dye assay on the scaffolds have found to be biocompatible and non-cytotoxic. These bioactive scaffolds can bring numerous applications for bone tissue repairs and regenerations.

## 1. Introduction

Bone tissue engineering is an advanced approach to treat bone defects caused by disease, aging, and accident by using fabricated scaffolds. It has brought numerous new biomaterials and methods developments for treating difficult segmental and contained skeletal defects. This concept has applied through the medicinal concept with principles, technologies, and methodologies of life sciences to repair, improve, and maintain their function [1,2,3,4]. Porous scaffolds with appropriate mechanical strength and biological properties, such as non-toxic, biocompatible, and biodegradable have played vital roles in cell adherence, proliferation, and growth of cells or tissues in bone repair [5,6,7]. The biodegraded scaffold should not elicit any toxic, harmful, damaging, or immunological response to the living tissues or organs [8,9,10].

The bone composition is comprised of organic and inorganic phases and made up of proteoglycans, glycosaminoglycans, glycoprotein collagen, and calcium phosphate [11,12,13,14]. Bone also contains an extraordinary amount of hydroxyapatite (HAp), which has suitable bioactive, biocompatible, and osteoconductive biological behavior. [15,16]. The brilliant biochemical affinity of HAp with natural bone tissue makes it a potential candidate for bone tissue engineering. Although having an excellent biological response, HAp is an inherently brittle material. The poor mechanical strength can be overcome by the use of polymers, creating a composite scaffold of polymer–ceramic [17,18]. Furthermore, antimicrobial activity of the scaffolds can be enhanced using a silver dip-coating method. Silver (Ag) is a suitable material for biological activities because of its anti-microbial and biocompatible properties [19,20]. Numerous research studies have reported their antimicrobial activities for series of various bacterium [21]. Therefore, silver has gained popularity as a popular antimicrobial material in biomedical and clinical applications. Insofar, silver-based composite materials are being used in the prevention of bacterial infection in orthopedics and dental implants [22]. Marsich et al. has reported alginate/HAp nanocomposite for bone graft with anti-bacterial activities due to the presence of Ag-nanoparticles and it was evident to be a suitable candidate for tissue engineering [23]. Jaiswal et al. used Ag-nanoparticles over titanium implant surface using immobilized Ag-nanoparticles and carried out osteoblast and anti-bacterial activities and found it to be antibacterial and osteogenic [24]. Das et al. fabricated Ag-coated nanotubes composite materials to increase osteogenesis and antimicrobial activities [25]. Tyllianakis et al. reported synthesized Ag-coated titanium-based nanocrystal to enhance antimicrobial activities [26]. Lee et al. fabricated porous scaffolds from gelatin and (1→3), (1→6)-*β*-glucan using the freeze-drying method. James et al. prepared carbon HAp/β-glucan composite for bone tissue engineering with enhanced biocompatibility [27]. The biopolymeric scaffolds have gained popularity over the decades. The scaffolds porosity and microstructure can be controlled using the types and amount of biopolymers [28,29]. β-glucan (BG) is a polysaccharide similar to the backbone of arabinoxylan or xyloglucan [30]. BG has promising biological properties like biocompatibility, biodegradability, and water adsorbing properties. It has potential applications in pharmaceutical and as well as other biomedical applications like drug carriers, wound healing, and tissue engineering [31,32]. 

However, the scaffold should be highly porous with interconnected pores to have enough space for cell adhesion, growth, differentiation, and migration [33]. In some instances, scaffolds contain bioactive substances with control release to affect incorporated cells. Different methods have been established to fabricate porous, three dimensional, and biodegradable scaffolds for bone tissue engineering like electrospinning, phase separation, gas-forming foam, thermal-induced separation, and freeze-drying [28,34]. 

The current research has focused on fabricating the synthesis of bioactive nanocomposite scaffolds (BNSs) for bone tissue engineering. Hence, β-Glucan, acrylic acid-based grafted and HAp was synthesized through free-radical polymerization, and scaffolds were prepared using the freeze-dried method. Then, the Ag dip-coating method was utilized to coat bioactive scaffolds with various concentrations of Ag. According to the best of our knowledge, this approach of fabricating Ag-coated bioactive scaffolds has not been reported thus far. Ag-coated bioactive nanocomposite scaffolds have potential antimicrobial and biocompatible properties that support cell adhesion, proliferation, and cell migration for osteogenesis. The structural, and morphological characteristics of these BNSs were studied using Fourier transform infrared spectroscopy (FTIR), X-ray diffraction analysis (XRD), energy-dispersive X-ray spectroscopy (EDS), scanning electron microscopy (SEM), and Brunauer–Emmett–Teller (BET). The mechanical properties were tested using the universal testing machine (UTM). The mouse pre-osteoblast cell line (MC3T3-E1) was used to investigate the biocompatibility of the scaffolds

## 2. Materials and Method

### 2.1. Materials

The ®-Glucan (BG) was purchased from DSP Gokyo Food and Chemical Co. Ltd., Osaka Japan. Acrylic acid (AAc) and *N,N’*-methylene-bis-acrylamide (*N*, N-MBA), n-HAp powder (<100 nm particle size), and AgNO_3_ were purchased from Sigma-Aldrich, Selangor, Malaysia. The phosphate buffer saline (PBS) solution was prepared using sodium hydrogen phosphate, potassium chloride, sodium chloride, di-potassium hydrogen phosphate, and hydrochloric acid obtained from Merck, Darmstadt, Germany and used without any purification.

### 2.2. Bioactive Nanocomposite Scaffold Fabrication

The biopolymeric nanocomposites were prepared by dissolving 2 g β-glucan (BG) in deionized-water and transferred into round bottom two-neck flask heated at 65 °C with constant stirring under an inert nitrogen atmosphere with addition of 0.05 g potassium persulfate as initiator. After 30 min, 0.40 mL of AAc as monomer and N, N-MBA crosslinker (0.05 % of AAc) were added into reaction media. After 45 min, (2.5 g) n-HAp powder was added gradually with continuous stirring for 3 h. Hence, grafting of acrylic acid into BG was done using free-radical polymerization. HAp nanoparticles have been trapped into the polymeric network of BG-graft-AAc on completion of the reaction. N_2_ gas was removed from the reaction media, followed by cooling at ambient. The reaction suspension was filtered under vacuum to remove unreacted reactants and washed with deionized water later it dried at 55 °C overnight in the oven. The synthesis of bioactive nanocomposite powder and their name identification are presented in Table 1. The bioactive nanocomposite powder (5 g) was homogenized into deionized-water (10 mL) to form slurry. Then slurry was filled into cylindrical molds (2 cm × 6 cm) and kept at −40 °C for 24 h. The porous bioactive nanocomposite scaffolds were obtained by and freeze drying method. The porosity and pore size of the bioactive nanocomposite scaffolds were analyzed using Brunauer–Emmett–Teller (Micromeritics Gemini II 2370) given in Table 1.

### 2.3. Deposition of Silver Particles on Bioactive Nanocomposite Scaffolds

**Figure 1:**
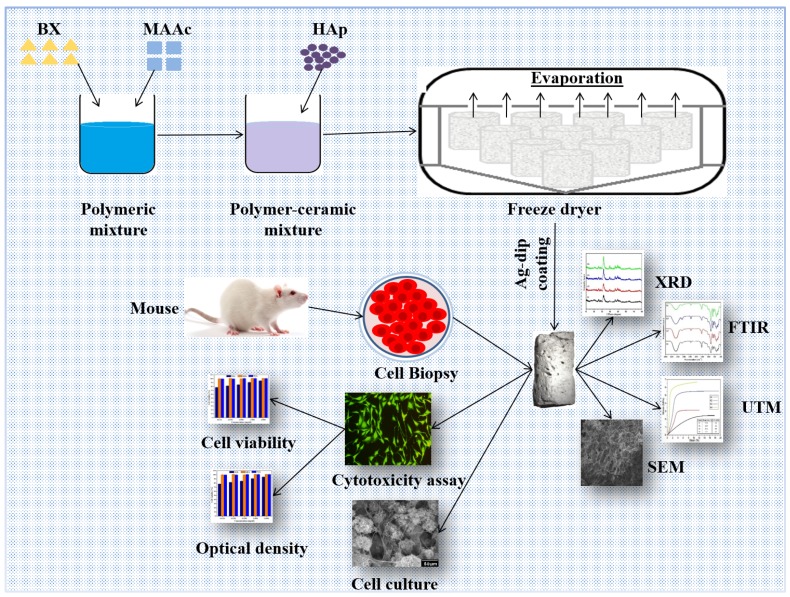
Schematic diagram of the preparation and characterization of a bioactive nanocomposite scaffold (BNS).

The bioactive scaffolds were washed with deionized water after treating with 10 wt. % NaOH_aq_. The solution was kept at ambient for 5 min. Initially, different concentrations of silver nitrate (AgNO_3_) solution was prepared from 0.15 to 0.60 M. Dropwise aqua ammonia (25 wt. %) was added into AgNO_3_ solutions. It was stirred to obtain a clear [Ag(NH_3_)_2_]^+^ solution. Then, scaffolds were dipped into the [Ag(NH_3_)_2_]^+^ solution for 20 s and oven-dried for 5 min at 100 °C. This process was repeated for 50 cycles to deposit Ag onto the scaffolds. Finally, Ag-coated bioactive nanocomposite scaffolds (BNS) were washed and dried as shown in the Figure 1.

## 3. Characterizations

### 3.1. Fourier Transform Infrared Spectroscopy

Chemical composition of BNS was assessed using FTIR (Shimadzu FTIR-8100A, Tokyo, Japan) with a resolution of 4 cm^−1^ and a frequency region from 400 to 4000 cm^−1^, and 16 scans were accumulated per sample. The samples were reduced to powder and analyzed as potassium bromide (KBr) pellets.

### 3.2. X-ray Diffraction

Phase analysis of the BNS was studied using Advance X-ray diffractometer (Bruker AXS D8, Kontich, Belgium) spectrometry. The XRD analysis was conducted through a diffractometer at a voltage of 40 kV and a current of 40 mA with Cu Kα radiation (1.540 Å) at 2θ range of 10–80° to determine the structure and peak data.

### 3.3. Scanning Electron Microscope/ Energy Dispersive Spectroscopy

Morphology of the BNS was observed by SEM (JEOL-JSM-6480, Peabody, Massachusetts, USA) coupled with EDS for elemental quantitative analysis. The samples were gold-sputtered. 

### 3.4. Mechanical Testing

Compression strength of the BNS was analyzed in a dry state by UTM (Testometrics, Rochdale, UK) with a compression loading rate of 5 mm/min. All cylindrical BNS (4 mm height and 10 mm diameter) were analyzed until failure. The elastic modulus was determined using the slope of the stress-strain curve. Five replicates per sample were tested.

### 3.5. Swelling Analysis

Scaffold swelling analysis was analyzed using water and phosphate buffer saline (PBS) solution by maintaining 7.4 pH at a temperature of 37 °C. The well dried scaffolds were soaked in solutions by keeping the conditions as mentioned above. The scaffolds were withdrawn from the solutions at different time periods and additional solvent concentrations were eliminated to have exact weight. The percentage of scaffold swelling has been estimated by Equation (1).

(1) $$Swelling\left(\%\right)=\frac{W_{S}-W_{D}}{W_{D}}\times 100$$

where, *Ws* = weight of swelling scaffolds and *W_D_* = weight of dried scaffolds at various time intervals.

### 3.6. In Vitro Studies

#### 3.6.1. Anti-Microbial Activities

An in vitro antimicrobial activity assay was conducted by agar disc-diffusion assay using gram-negative model bacterium *Escherichia coli* DH5 alpha. These bacterial strains were incubated at 37 °C to analyze antimicrobial activities of materials. Bacterial culture was spread uniformly using sterile glass rod over solidified agar [35]. Then 90 mL of each scaffold extract was placed over the bacterial Petri-plate. The Petri-plate was kept into an oven incubated for 24 h at 37 °C.

#### 3.6.2. Sample Preparation for Cell Culture

BNS3 extract was selected for cell culture observation. The bottom of each well of 24-well plate was finely coated with scaffold and UV-light sterilized for 1 h and used to study morphological changes of the cells. Different concentrations of BNS3 extract from 0.125 to 2.00 mg/mL were prepared to evaluate cell viability and uncoated wells were used as control.

#### 3.6.3. Cell Morphological Analysis

The MC3T3-E1 mouse pre-osteoblast cell line was purchased from American Type Culture Collection (ATCC, Manassas, VA, USA). Cells were cultured on coated wells of 24 well plates at the density of approximately 5000 cells per cm^2^ in α-MEM supplemented with 10% FBS (Fetal Bovine Serum Gibco™ 12662011, Gibco laboratories, Gaithersburg, MD, USA, 100 U/mL Penicillin and 0.1 mg/mL Streptomycin solution (Gibco™ 15140122, ATCC, Manassas, VA, USA). The cells were incubated for 72 h in 5% CO_2_ with 90% humidity at 37 °C. The cell morphology was analyzed using a Nikon *ECLIPSE* TS100 (ATCC, Manassas) inverted fluorescence microscope with live cells stained using 10 µg/mL of Fluorescein diacetate solution in complete growth medium to minimize the background scaffold coating and highlight only living viable cells under 488 nm excitation wavelength.

#### 3.6.4. Cytotoxicity Using the Neutral Red Assay

The pre-osteoblast cell viability assay was performed by seeding cells in a 12-well plate with approximately 5,000 cells per cm^2^ for 24h. Different concentrations of BNS3 extract (from 0.125 to 2.00 mg mL^−1^), dimethyl sulfoxide (DMSO) (1%) and non-treated cells were taken as negative and positive control, respectively. The neutral red assay of cells was performed after 24, 48, and 72 h, reported by Repetto [36]. The treated and control cells were incubated in 40 µg/mL neutral red in complete growth medium for 2 h and washed with phosphate buffer saline (BS). Picked up dye was released in a de-staining solution consisting of 1% glacial acetic acid, 49% distilled water, and 50% ethanol for 5 min at room temperature. The optical density was measured at 540 nm using a spectrophotometer and cell viability (%) by Equation (2).

(2) $$\mathrm{Cell}\;\mathrm{viability}\;\left(\%\right)=\frac{{\mathrm{OD}}_{S}}{{\mathrm{OD}}_{C}}\times 100$$

whereas OD_S_ represents Optical Density in treated samples, and OD_C_ designates Optical Density in positive control.

#### 3.6.5. SEM Sample Preparation

The images of the cultured cell on BNS3 were taken using SEM (JSM 6940A, Jeol, Tokyo, Japan) with a different period; 24 h, 48 h, 72 h, and 7 days. The cultured cells were washed with PBS and fixed by absolute ethanol for 7 min and kept at 4 °C. Before SEM analysis, the well dried samples were gold-sputtered and analyzed with 1 kV voltage, 7 × 10^−2^ bar pressure and 20 mA deposition current for 2.0 min.

#### 3.6.6. Statistical Analysis

The quantitative data were obtained in triplicate form and expressed as the mean ± standard deviation (SD). The statistical analysis conducted using statistical analysis system software (IBM SPSS Statistics 21, SPSS Inc., New York, NY, USA) and value of *p* < 0.05 and *n* = 3 taken as statistically significant.

## 4. Results and Discussion

The freeze-dried porous BNS samples have been prepared using n-HAp in the grafted natural polymer. The acrylic acid was grafted into β-glucan through the free-radical polymerization process and, n-HAp has been trapped into the polymeric matrix of grafted BG during the reaction.

### 4.1. FTIR

**Figure 2:**
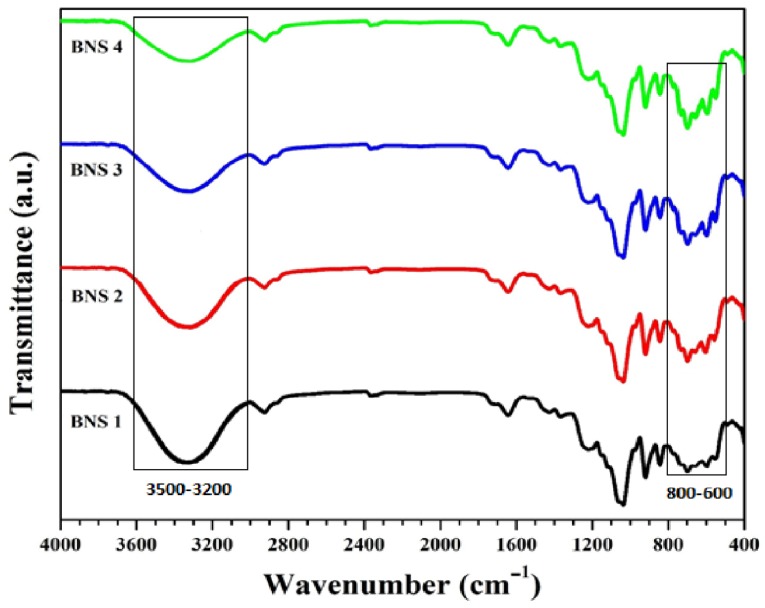
Fourier transform infrared spectroscopy (FTIR) spectral profiles show various functional groups along with their different modes of vibrations available in the BNS.

Figure 2 shows the spectral peaks at 1093 cm^−1^ are described triply degenerated P-O stretching [37]. Whereas, peaks at 603 and 569 cm^−1^ describes the bending mode of O-P-O. The absorption band in the region from 560 to 600 and from 1000 to 1100, cm^−1^ were attributed to the presence of calcium phosphate moiety of HAp [37,38]. Hence, presence of n-HAp has been confirmed by PO_4_^−3^ at 630 cm^−1^ into BNS [39,40,41]. The peak at 1220 cm^−1^, 906 cm^−1^, and 1033 cm^−1^ attributed to C–O cyclic, pyranose, and functional group of acrylic. These vibrations might due to the formation of covalent bond between BG and AAc [42]. The band at 1740 cm^−1^ corresponds to the stretching vibration of AAc carbonyl group. The peaks/bands between 1430 and 1450 cm^−1^ are the result of C–O stretching and C–O–H bending vibrations. Consequently, the presence of all these peaks/bands and the demise of BG’s O–H bending vibration is confirmation that AAc grafted the BG polysaccharide on the O–H site. These peaks/bands confirm the grafting of AAc on the backbone of the polysaccharide [43]. The adsorption peak at 947 cm^−1^ is due to C–O stretching [44]. Stretching vibration at 412 cm^−1^ is the characteristic of silver and hydroxyl (OH^−^) has a steric effect on coordination between oxygen (O) and Ag-particles, the electronegativity of oxygen is higher due to its donating ability [45]. The absorption bands from 3600 to 3100 cm^−1^ (Figure 2) attribute to the hydroxyl stretching vibrations. The absorption at 2950–2850 cm^−1^ expresses the aliphatic C-H stretching vibration.

### 4.2. XRD

**Figure 3:**
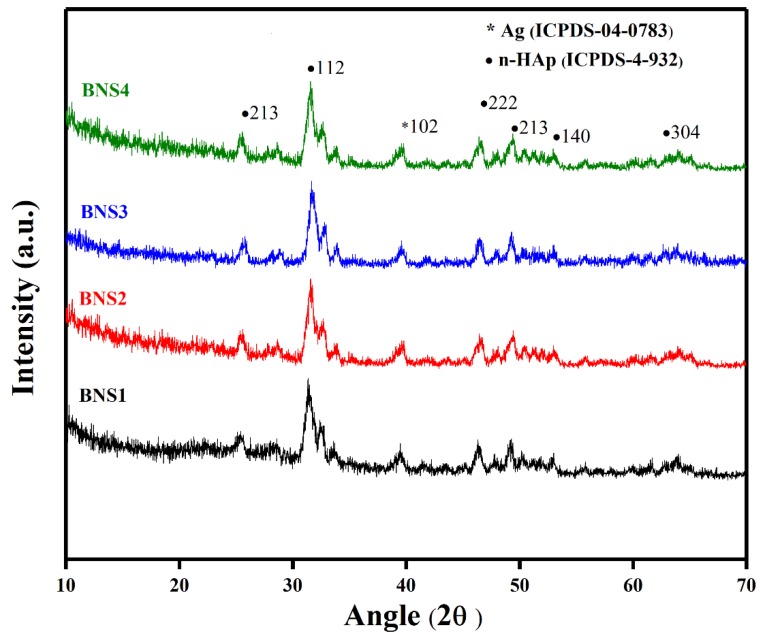
X-ray diffraction (XRD) spectra of BNS samples.

The XRD pattern is shown in Figure 3 for organic and inorganic phase analysis of the BNS. The low intensity of organic substances (BG and AAc) has exhibited at a characteristic peak near 2θ of 26° with reduced crystallinity [46]. This reduced crystallinity is due to the H-bond formation and chemical reaction of BG and AAc that results in reduced crystallinity of BG in BNS (observed in FTIR Spectra). The presence of the n-HAp into the polymeric matrix of grafted BG leads towards non-crystalline behavior of the polymeric region [47,48]. The observed sharp peaks of HAp are at an angle of 2ѳ of 25.3°, 31.4°, 46.6°, 49.6°, 51.3°, and 61.3° (ICPDS-04-0783) with their corresponding diffraction planes of (213), (112), (222), (213), (140), and (304) as shown in Figure 3. The peak at 39.69° indicates the existence of Ag-coated with corresponding (102) diffraction plane which matched with standard card (ICPDS-04-932). Furthermore, the calculated cell parameters BNS are (a = b = 9.4000) and (c = 6.9300), which is perfectly match with standard data (PDF-4-932). The detailed calculated parameters of prepared samples shown in the Table 2 below.

### 4.3. SEM–EDX

**Figure 4:**
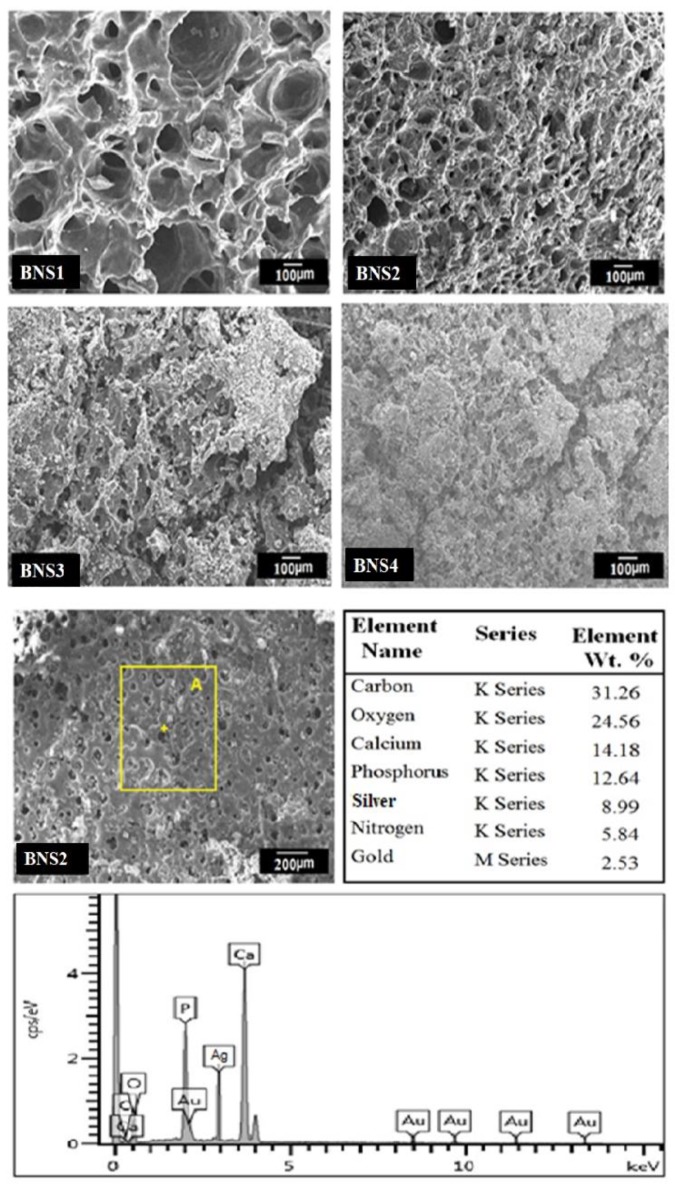
Scanning electron microscopy (SEM) images of BNS samples and elemental analysis of BNS3.

The SEM analysis has been performed to investigate the pore size and surface morphology of BNS, as depicted in Figure 4. All of the BNS samples show porous and rough surface morphology. The irregular, non-smooth, and rough surface morphology help cell adhesion, while the porosity with appropriate pore size encourages cell proliferation and adhesion [49,50]. Additionally, the SEM profile has presented a different pore size to promote cell adhesion, proliferation, diffraction, and extracellular matrix secretion. The BNS almost have an almost uniform pore size from 83 ± 2 μm to 175 ± 2 μm, whereas, BNS3 had 108 ± 1 μm with an average pore size suitable for osteointegration. However, 50–200 m is an appropriate pore size for osteoblast cell attachment, proliferation [51,52]. Hence, BNS2 with proper pore size has encouraged cell proliferation and cell culture (evident from Figure 9). The conducted elemental analysis of the bioactive scaffold confirmed the presence of HAp by available carbon (31.26%), oxygen (24.56%), calcium (14.18%), phosphorus (12.64%), and silver (8.99%) into BNS3 as shown Figure 4 The overall SEM–EDX results like: morphology, pore size, porosity, and available elements show that these scaffolds are helpful to regenerate and have vital role for bone tissue engineering [16]. 

### 4.4. Mechanical Testing

**Figure 5:**
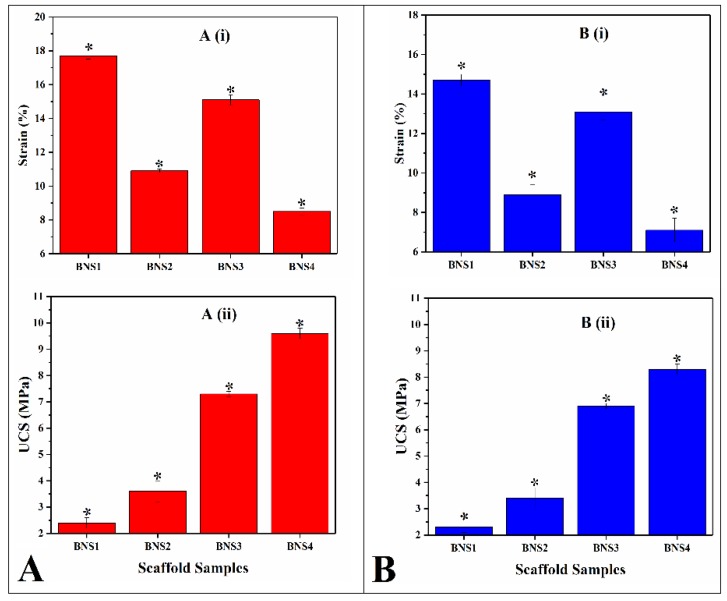
Mechanical behavior of all scaffold samples in dry form A and wet form B at room temperature: (**A**,**B**) (i) percentage of strain (strain %) of all BNS samples. (**A**,**B**) (ii) unconfined compression strength testing (UTS) of all BNS samples.

Mechanical strength of all BNS samples have been studied in dry and wet form as shown in Figure 5. Deionized water (H_2_O) was used as media used for wet analysis. The coating concentrations of AgNO_3_ have changed the mechanical feature of the BNS. It was found that increasing concentrations leads to enhance compressive strength. It was also found that mechanical strength of these scaffold samples and exhibited lower mechanical strength in wet environment due to release of Ag-particle into aqueous media [53,54]. Ag-coating has a vital role in improving the mechanical strength of BNS samples with suitable porosity for tissue engineering [55]. The polymeric matrix has a different chemical structure and impact over the grain boundary. Hence, Ag-coating has the capability of inducing functional properties like porosity, pore size, grain boundary, which also affects the mechanical strength [56].

### 4.5. Swelling Analysis

The swelling behavior of scaffold has a vital role in biological activities like in vitro studies. The body fluid contains cell nutrients and metabolites that absorbs during swelling of scaffold. More is the swelling and more transfer of body fluids. The porosity, pore size, and surface area of scaffolds increases due to swelling that helps cell migration, adhesion, and proliferation [57,58]. Moreover, controlled swelling of scaffolds under physiological conditions strengthen the scaffolds structure with controlled degradation that plays a key role for bone regeneration [59].

**Figure 6:**
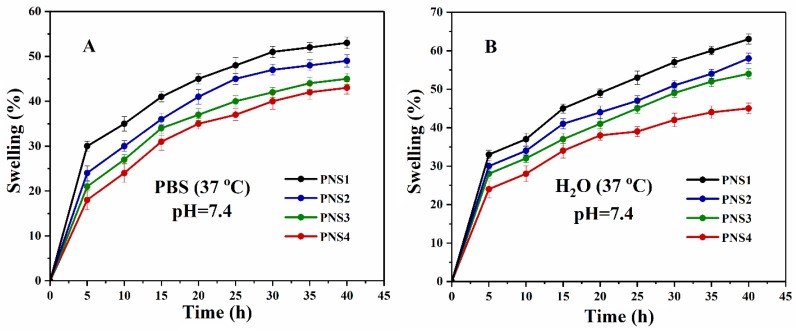
Swelling behavior of scaffolds in phosphate buffer saline (PBS) (**A**) and water (**B**) keeping temperature at 37 °C and 7.4 pH.

The swelling effects of scaffolds were analyzed under various physiological conditions, such as temperatures, pH levels, and time intervals. Figure 6A,B shows that these scaffolds exhibited different rate of swelling in aqueous media and PBS solution at a temperature of 37 °C and pH of 7.4 and continued to swell until equilibrium. At these conditions, all scaffolds were showing different increasing rate of swelling. The highest and lowest swelling rate were observed for PNS1 and PNS4, respectively. The different swelling behavior of these scaffolds was due to coating of various silver concentrations that cause increase or decrease in ionic swelling pressure in both media [60]. Swelling rate of any material mainly depends upon the osmotic pressure. The increasing concentration of silver solution also responsible in decreasing rate of swelling [61]. The swelling of these scaffolds are different in aqueous media and PBS solution due to different hydrogen bonding, hydrophilic and hydrophobic interactions, and other forces of attraction that cause different osmotic pressure [62]. So, there is an inverse relation between silver concentration and rate of swelling.

### 4.6. In-Vitro Study

#### 4.6.1. Anti-Microbial Activity

**Figure 7:**
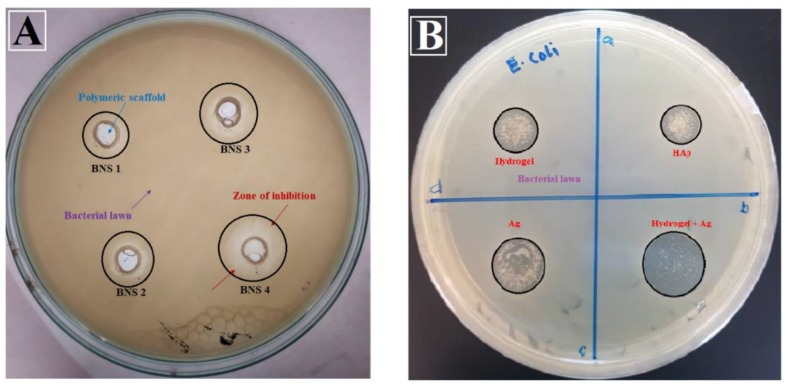
Anti-microbial activities of all scaffold samples (**A**) and individual materials (**B**) against *E. coli*.

Antimicrobial activities of BNS samples (A) and individual materials (B) were studied using agar well-diffusion assay against *E. coli* DH5 alpha. It was found that silver has an important role in antimicrobial activities as shown in Figure 7A,B [63]. The anti-microbial activity of silver nanoparticles attributes was due to adsorption into the cell wall and interaction with cellular protein. Denaturation of proteins was a result of sliver-nanoparticle permeability and accretion of an external fluid, owing to better antimicrobial activities of Ag-coated scaffolds [64]. The charged component of the bacterial surface membrane, i.e., phospholipids and lipopolysaccharides interacted with several available functional groups of BNS silver nanoparticles [65,66]. The active sites of hydrogels and nano-Ag endorsed their changes into the bacteria membrane, hindering the microbe’s activity. The bacterial growth was also delayed due to the bonding between BG and bacterial DNA, thus leading to inhibition of bacterial transcription and translation. The molecular alteration in DNA due to Ag-coated bioactive scaffolds resulted in little or no growth of bacteria. Hence, the sample BNS4 showed maximum antimicrobial activity that is due to higher concentration of Ag-coating over the scaffold. 

#### 4.6.2. Cytotoxicity

**Figure 8:**
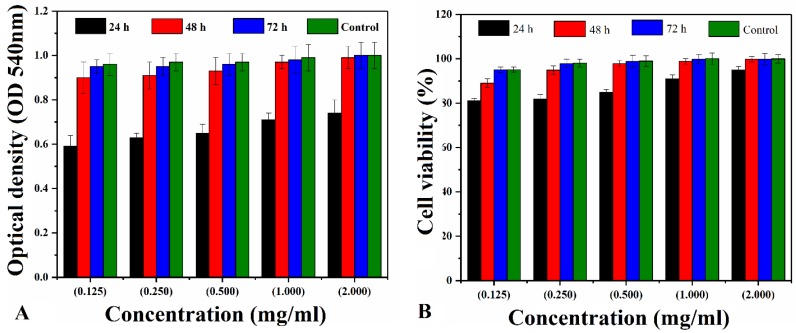
Different concentrations of extract were used to estimate percentage cell viability on mouse preosteoblast cell line MC3T3-E1 for 24, 48, and 72 h of time points. (**A**) shows the optical density of absorbed neutral red dye at 540 nm while (**B**) represents the relative percentage of cells compared to control.

BNS3 has been selected due to its adequate porosity and compressive strength for biological activities. Figure 8 shows the cytotoxicity response of the bioactive nanocomposite scaffold against different concentrations (from 0.125 to 2.00 mg mL^−1^) of BNS3 extract against MC3T3-E1 cell line. Considering the control cell viability as 100% the cell viability of bioactive scaffolds was relatively calculated. The BNS3 has shown excellent cell viability with only 6–15% cell loss in the process.

#### 4.6.3. Morphological Changes and Cell Attachment

**Figure 9:**
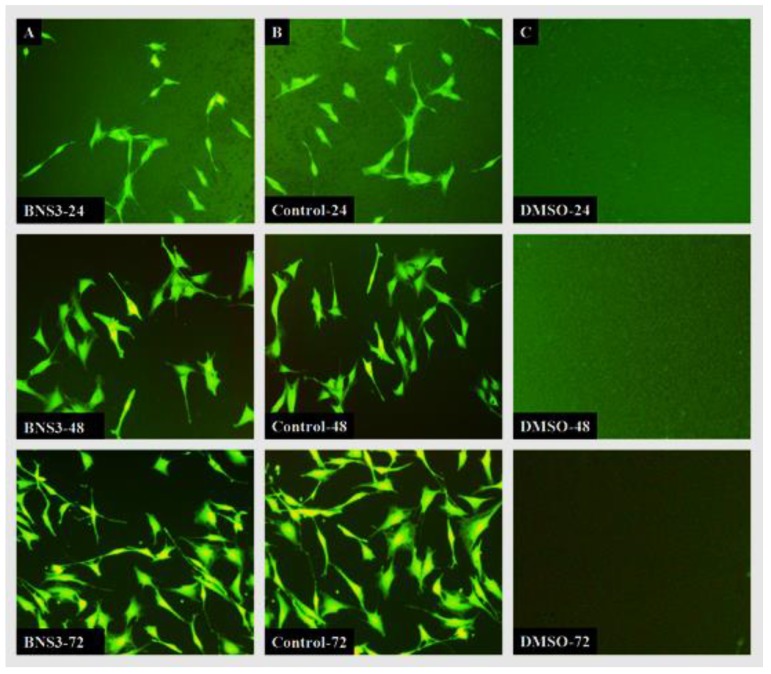
Morphological changes in cells observed through attached cells on the coated surface. Untreated but tissue culture treated wells without any additional coating material used as positive control wells. 1% DMSO as a negative control. (**A**) Sample BNS 3; (**B**) positive control; (**C**) negative control at 24, 48 and 72h.

Figure 9 shows the cell morphology and adhesion against BNS3 observed under the fluorescent microscope. The cell attachment pattern in BSN3 coated wells, and positive control wells were alike, and the proliferation rate with each successive day also appeared similar. Moreover, cell morphology in coated wells appeared as cylindrical as in positive control wells. After 72 h, cells started gaining standard fibroblast-like morphology with extended cell bodies showing sufficient cell attachment over the coated surface. It was observed that cells attached have the same morphology as for all time points, while DMSO treated wells do not have any cells attached.

#### 4.6.4. SEM Analysis of Cell Growth

**Figure 10:**
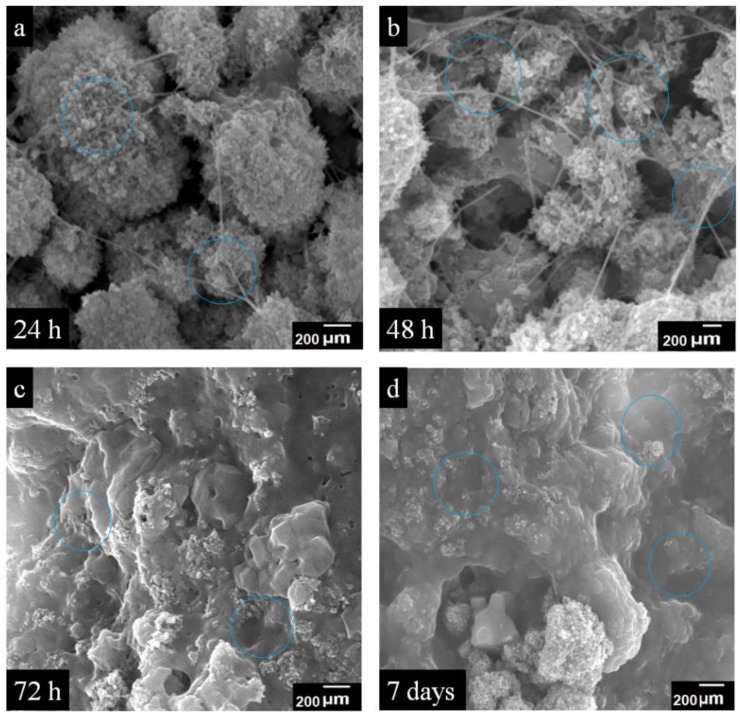
SEM images of pre-osteoblast over BNS3 after: (**a**) 24 h, (**b**) 48 h, (**c**) 72 h, and (**d**) 7 days of cell growth.

Figure 10 presents SEM analysis of cell culture over the BNS at 200 µm magnification and blue circles indicate the cell culture area. The MC3T3-E1 cell-line used for cell growth analysis over BNS3 along with and taking Themanox TM substrate as a positive control. Subsequently, the specific incubation time (48 h, 72 h, and 7 days), the pre-osteoblast cells adhered and proliferated over BNS3 by showing bioactive behavior, which spread all into and over the porous surface of BNS3. The SEM has been used to analyze the attachment and cell culture of the MC3T3-E1 cell line and expressed through images: (a) after 24 h, (b) after 24 h (c) after 72 h, and (d) after 7 days of cell culture, respectively.

## 5. Conclusions

We have fabricated Ag-coated BG/n-Hap bioactive nanocomposite scaffold via the freeze-dried method to mimic the functionalization of the extracellular matrix for bone tissue engineering. Ag-coated scaffolds are mechanically robust and have sufficient compression strength near to the cancellous bone. Higher concentration of Ag will decrease the pore size but elevate the mechanical strength and anti-microbial activities of BNS samples. The biological studies have confirmed that BNS3 has potential for growth of the MC3T3-E1 cell line by maintaining their cell density without compromising the cell viability. Hence, the presented BNS samples have potential applications for bone tissue engineering.

## Figures and Tables

**Table 1:** Composition of the bioactive nanocomposite scaffolds, their respective porosity, and pore size.

Samples	AgNO_3_ Concentration (M)	Pore Size (µm)	Porosity (%)
BNS1	0.15	175 ± 2	88.5 ± 2
BNS2	0.30	135 ± 1	81.5 ± 1
BNS3	0.45	115 ± 2	74.5 ± 2
BNS4	0.60	92 ± 2	69.5 ± 1

**Table 2:** Calculated parameters of prepared BNS samples.

Sample		Angle (2ѳ)		FWMH		Lattice Strain (%)		d (Å)		D (nm)	
**BNS1**		31.1°	39.69°	0.70317	0.94232	0.0218	0.0115	3.620	2.45	12.25	9.36
**HAp**	**Ag**										
**BNS2**		31.1°	39.69°	0.80141	0.96215	0.0208	0.0232	3.45	2.41	12.39	9.74
**HAp**	**Ag**										
**BNS3**		31.1°	39.69°	0.8316	0.97137	0.0148	0.0212	3.36	2.32	12.95	9.82
**HAp**	**Ag**										
**BNS4**		31.1°	39.69°	0.90317	0.99182	0.0217	0.0315	3.14	2.25	13.35	9.96
**HAp**	**Ag**										

The average crystallite size of 49.9 nm.

## References

[1] Nettles D.L., Elder S.H., Gilbert J.A. (2002). Potential use of chitosan as a cell scaffold material for cartilage tissue engineering. Tissue Eng..

[2] Salgado A.J., Coutinho O.P., Reis R.L. (2004). Bone tissue engineering: State of the art and future trends. Macromol. Biosci..

[3] Ramseier C.A., Rasperini G., Batia S., Giannobile W.V. (2012). Advanced reconstructive technologies for periodontal tissue repair. Periodontology.

[4] Wang W., Liu Y., Yang C., Qi X., Li S., Liu C., Li X. (2019). Mesoporous bioactive glass combined with graphene oxide scaffolds for bone repair. Int. J. Biol. Sci..

[5] Lasprilla A.J., Martinez G.A., Lunelli B.H., Jardini A.L., Maciel Filho R. (2012). Poly-lactic acid synthesis for application in biomedical devices—A review. Biotechnol. Adv..

[6] Arabnejad S., Johnston R.B., Pura J.A., Singh B., Tanzer M., Pasini D. (2016). High-strength porous biomaterials for bone replacement: A strategy to assess the interplay between cell morphology, mechanical properties, bone ingrowth and manufacturing constraints. Acta Biomater..

[7] Quinlan E., López-Noriega A., Thompson E., Kelly H.M., Cryan S.A., O’Brien F.J. (2015). Development of collagen–hydroxyapatite scaffolds incorporating PLGA and alginate microparticles for the controlled delivery of rhBMP-2 for bone tissue engineering. J. Control. Release.

[8] Elkhenany H., Amelse L., Lafont A., Bourdo S., Caldwell M., Neilsen N., Dervishi E., Derek O., Biris A.S., Anderson D. (2015). Graphene supports in vitro proliferation and osteogenic differentiation of goat adult mesenchymal stem cells: Potential for bone tissue engineering. J. Appl. Toxicol..

[9] Hussein K.H., Park K.-M., Kang K.-S., Woo H.-M. (2016). Biocompatibility evaluation of tissue-engineered decellularized scaffolds for biomedical application. Mater. Sci. Eng. C.

[10] Singh R.S., Kaur N., Rana V., Kennedy J.F. (2016). Recent insights on applications of pullulan in tissue engineering. Carbohydr. Polym..

[11] Rezwan K., Chen Q., Blaker J., Boccaccini A.R. (2006). Biodegradable and bioactive porous polymer/inorganic composite scaffolds for bone tissue engineering. Biomaterials.

[12] Li Z., Ramay H.R., Hauch K.D., Xiao D., Zhang M. (2005). Chitosan–alginate hybrid scaffolds for bone tissue engineering. Biomaterials.

[13] Ansari S., Khorshidi S., Karkhaneh A. (2019). Engineering of gradient osteochondral tissue: From nature to lab. Acta Biomater..

[14] Ma G., Yang D., Su D., Mu X., Kennedy J.F., Nie J. (2010). Preparation and properties of water-soluble chitosan and polyvinyl alcohol blend films as potential bone tissue engineering matrix. Polym. Adv. Technol..

[15] Fathi M., Hanifi A., Mortazavi V. (2008). Preparation and bioactivity evaluation of bone-like hydroxyapatite nanopowder. J. Mater. Process. Technol..

[16] Mondal S., Hoang G., Manivasagan P., Moorthy M.S., Nguyen T.P., Phan T.T.V., Kim H.H., Kim M.H., Nam S.Y., Oh J. (2018). Nano-hydroxyapatite bioactive glass composite scaffold with enhanced mechanical and biological performance for tissue engineering application. Ceram. Int..

[17] Ramesh N., Moratti S.C., Dias G.J. (2018). Hydroxyapatite–polymer biocomposites for bone regeneration: A review of current trends. J. Biomed. Mater. Res. Part B Appl. Biomater..

[18] Khoshroo K., Kashi T.S.J., Moztarzadeh F., Tahriri M., Jazayeri H.E., Tayebi L. (2017). Development of 3D PCL microsphere/TiO2 nanotube composite scaffolds for bone tissue engineering. Mater. Sci. Eng. C.

[19] Shankar S., Jaiswal L., Aparna R., Prasad R. (2014). Synthesis, characterization, in vitro biocompatibility, and antimicrobial activity of gold, silver and gold silver alloy nanoparticles prepared from Lansium domesticum fruit peel extract. Mater. Lett..

[20] Wu X., Li J., Wang L., Huang D., Zuo Y., Li Y. (2010). The release properties of silver ions from Ag-nHA/TiO2/PA66 antimicrobial composite scaffolds. Biomed. Mater..

[21] Li Y., Luo Y., Hu Y., Zhu D.-D., Zhang S., Liu Z.-J., Gong H.-B., Zhu H.-L. (2012). Design, synthesis and antimicrobial activities of nitroimidazole derivatives containing 1, 3, 4-oxadiazole scaffold as FabH inhibitors. Bioorganic Med. Chem..

[22] Travan A., Marsich E., Donati I., Benincasa M., Giazzon M., Felisari L., Paoletti S. (2011). Silver–polysaccharide nanocomposite antimicrobial coatings for methacrylic thermosets. Acta Biomater..

[23] Marsich E., Bellomo F., Turco G., Travan A., Donati I., Paoletti S. (2013). Nano-composite scaffolds for bone tissue engineering containing silver nanoparticles: Preparation, characterization and biological properties. J. Mater. Sci. Mater. Med..

[24] Jaiswal S., McHale P., Duffy B. (2012). Preparation and rapid analysis of antibacterial silver, copper and zinc doped sol–gel surfaces. Colloids Surf. B Biointerfaces.

[25] Das K., Bose S., Bandyopadhyay A., Karandikar B., Gibbins B.L. (2008). Surface coatings for improvement of bone cell materials and antimicrobial activities of Ti implants. J. Biomed. Mater. Res. Part B Appl. Biomater..

[26] Tyllianakis M., Dalas E., Christofidou M., Kallitsis J., Chrissanthopoulos A., Koutsoukos P., Bartzavali C., Gourdoupi N., Papadimitriou K., Oikonomou E. (2010). Novel composites materials from functionalized polymers and silver coated titanium oxide capable for calcium phosphate induction, control of orthopedic biofilm infections: An “In Vitro” study. J. Mater. Sci. Mater. Med..

[27] Sroka-Bartnicka A., Kimber J.A., Borkowski L., Pawlowska M., Polkowska I., Kalisz G., Belcarz A., Jozwiak K., Ginalska G., Kazarian S.G. (2015). The biocompatibility of carbon hydroxyapatite/β-glucan composite for bone tissue engineering studied with Raman and FTIR spectroscopic imaging. Anal. Bioanal. Chem..

[28] Wei G., Ma P.X. (2004). Structure and properties of nano-hydroxyapatite/polymer composite scaffolds for bone tissue engineering. Biomaterials.

[29] Wu X., Liu Y., Li X., Wen P., Zhang Y., Long Y., Wang X., Guo Y., Xing F., Gao J. (2010). Preparation of aligned porous gelatin scaffolds by unidirectional freeze-drying method. Acta Biomater..

[30] Gibeaut D.M., Pauly M., Bacic A., Fincher G.B. (2005). Changes in cell wall polysaccharides in developing barley (Hordeum vulgare) coleoptiles. Planta.

[31] Regand A., Chowdhury Z., Tosh S.M., Wolever T.M., Wood P. (2011). The molecular weight, solubility and viscosity of oat beta-glucan affect human glycemic response by modifying starch digestibility. Food Chem..

[32] Choromanska A., Kulbacka J., Rembialkowska N., Pilat J., Oledzki R., Harasym J., Saczko J. (2015). Anticancer properties of low molecular weight oat beta-glucan–an in vitro study. Int. J. Biol. Macromol..

[33] Hutmacher D.W. (2000). Scaffolds in tissue engineering bone and cartilage. The Biomaterials: Silver Jubilee Compendium.

[34] Christenson E.M., Anseth K.S., van den Beucken J.J., Chan C.K., Ercan B., Jansen J.A., Laurencin C.T., Li W.J., Murugan R., Nair L.S. (2007). Nanobiomaterial applications in orthopedics. J. Orthop. Res..

[35] Alzoreky N., Nakahara K. (2003). Antibacterial activity of extracts from some edible plants commonly consumed in Asia. Int. J. Food Microbiol..

[36] Repetto G., Del Peso A., Zurita J.L. (2008). Neutral red uptake assay for the estimation of cell viability/cytotoxicity. Nat. Protoc..

[37] Anjaneyulu U., Pattanayak D.K., Vijayalakshmi U. (2016). Snail shell derived natural hydroxyapatite: Effects on NIH-3T3 cells for orthopedic applications. Mater. Manuf. Process..

[38] Destainville A., Champion E., Bernache-Assollant D., Laborde E. (2003). Synthesis, characterization and thermal behavior of apatitic tricalcium phosphate. Mater. Chem. Phys..

[39] Rouahi M., Gallet O., Champion E., Dentzer J., Hardouin P., Anselme K. (2006). Influence of hydroxyapatite microstructure on human bone cell response. J. Biomed. Mater. Res. Part A.

[40] Suchanek W.L., Byrappa K., Shuk P., Riman R.E., Janas V.F., TenHuisen K.S. (2004). Preparation of magnesium-substituted hydroxyapatite powders by the mechanochemical–hydrothermal method. Biomaterials.

[41] Furuzono T., Yasuda S., Kimura T., Kyotani S., Tanaka J., Kishida A. (2004). Nano-scaled hydroxyapatite/polymer composite IV. Fabrication and cell adhesion of a 3D scaffold made of composite material with a silk fibroin substrate to develop a percutaneous device. J. Artif. Organs.

[42] Kamal H., Abd-Elrahim F., Lotfy S. (2014). Characterization and some properties of cellulose acetate-co-polyethylene oxide blends prepared by the use of gamma irradiation. J. Radiat. Res. Appl. Sci..

[43] Srivastava A., Kumar R. (2013). Synthesis and characterization of acrylic acid-g-(-carrageenan) copolymer and study of its application. Int. J. Carbohydr. Chem..

[44] Sarker B., Papageorgiou D.G., Silva R., Zehnder T., Gul-E-Noor F., Bertmer M., Kaschta J., Chrissafis K., Detsch R., Boccaccini A.R. (2014). Fabrication of alginate–gelatin crosslinked hydrogel microcapsules and evaluation of the microstructure and physico-chemical properties. J. Mater. Chem. B.

[45] Askari M.B., Banizi Z.T., Seifi M., Dehaghi S.B., Veisi P. (2017). Synthesis of TiO2 nanoparticles and decorated multi-wall carbon nanotube (MWCNT) with anatase TiO2 nanoparticles and study of optical properties and structural characterization of TiO2/MWCNT nanocomposite. Opt. Int. J. Light Electron. Opt..

[46] Abd-Khorsand S., Saber-Samandari S., Saber-Samandari S. (2017). Development of nanocomposite scaffolds based on TiO2 doped in grafted chitosan/hydroxyapatite by freeze drying method and evaluation of biocompatibility. Int. J. Biol. Macromol..

[47] Laurencin C.T., Nair L.S. (2014). Nanotechnology and Regenerative Engineering: The Scaffold.

[48] Saber-Samandari S., Yilmaz O., Yilmaz E. (2012). Photoinduced graft copolymerization onto chitosan under heterogeneous conditions. J. Macromol. Sci. Part A.

[49] Ibrahim S., Sabudin S., Sahid S., Marzuke M., Hussin Z., Bashah N.K., Jamuna-Thevi K. (2016). Bioactivity studies and adhesion of human osteoblast (hFOB) on silicon-biphasic calcium phosphate material. Saudi J. Biol. Sci..

[50] Dick T., Dos Santos L. (2017). In Situ synthesis and characterization of hydroxyapatite/natural rubber composites for biomedical applications. Mater. Sci. Eng. C.

[51] Li X., Wang L., Fan Y., Feng Q., Cui F.Z., Watari F. (2013). Nanostructured scaffolds for bone tissue engineering. J. Biomed. Mater. Res. Part A.

[52] Oh S.H., Park I.K., Kim J.M., Lee J.H. (2007). In Vitro and in vivo characteristics of PCL scaffolds with pore size gradient fabricated by a centrifugation method. Biomaterials.

[53] Samanipour F., Bayati M., Zargar H., Golestani-Fard F., Troczynski T., Taheri M. (2011). Electrophoretic enhanced micro arc oxidation of ZrO2–HAp–TiO2 nanostructured porous layers. J. Alloy. Compd..

[54] Kaviyarasu K., Mariappan A., Neyvasagam K., Ayeshamariam A., Pandi P., Palanichamy R.R., Gopinathan C., Mola G.T., Maaza M. (2017). Photocatalytic performance and antimicrobial activities of HAp-TiO2 nanocomposite thin films by sol-gel method. Surf. Interfaces.

[55] Tamaddon M., Samizadeh S., Wang L., Blunn G., Liu C. (2017). Intrinsic Osteoinductivity of Porous Titanium Scaffold for Bone Tissue Engineering. Int. J. Biomater..

[56] Sionkowska A., Kaczmarek B. (2017). Preparation and characterization of composites based on the blends of collagen, chitosan and hyaluronic acid with nano-hydroxyapatite. Int. J. Biol. Macromol..

[57] Monfregola L., Bugatti V., Amodeo P., De Luca S., Vittoria V. (2011). Physical and water sorption properties of chemically modified pectin with an environmentally friendly process. Biomacromolecules.

[58] Mohan N., Nair P.D. (2005). Novel porous, polysaccharide scaffolds for tissue engineering applications. Trends Biomater. Artif. Organs.

[59] Kim C.H., Khil M.S., Kim H.Y., Lee H.U., Jahng K.Y. (2006). An improved hydrophilicity via electrospinning for enhanced cell attachment and proliferation. J. Biomed. Mater. Res. Part B Appl. Biomater..

[60] Nazemi K., Moztarzadeh F., Jalali N., Asgari S., Mozafari M. (2014). Synthesis and characterization of poly (lactic-co-glycolic) acid nanoparticles-loaded chitosan/bioactive glass scaffolds as a localized delivery system in the bone defects. BioMed Res. Int..

[61] Bennet D., Marimuthu M., Kim S., An J. (2012). Dual drug-loaded nanoparticles on self-integrated scaffold for controlled delivery. Int. J. Nanomed..

[62] Maji K., Dasgupta S., Pramanik K., Bissoyi A. (2016). Preparation and evaluation of gelatin-chitosan-nanobioglass 3D porous scaffold for bone tissue engineering. Int. J. Biomater..

[63] Feng Q.L., Wu J., Chen G., Cui F., Kim T., Kim J. (2000). A mechanistic study of the antibacterial effect of silver ions on Escherichia coli and Staphylococcus aureus. J. Biomed. Mater. Res..

[64] Yamanaka M., Hara K., Kudo J. (2005). Bactericidal actions of a silver ion solution on Escherichia coli, studied by energy-filtering transmission electron microscopy and proteomic analysis. Appl. Environ. Microbiol..

[65] Lu Y., Li X., Zhou X., Wang Q., Shi X., Du Y., Deng H., Jiang L. (2014). Characterization and cytotoxicity study of nanofibrous mats incorporating rectorite and carbon nanotubes. RSC Adv..

[66] Zykwinska A., Tripon-Le Berre L., Sinquin C., Ropartz D., Rogniaux H., Colliec-Jouault S., Delbarre-Ladrat C. (2018). Enzymatic depolymerization of the GY785 exopolysaccharide produced by the deep-sea hydrothermal bacterium Alteromonas infernus: Structural study and enzyme activity assessment. Carbohydr. Polym..

